# Short- and long-term normal tissue damage with photodynamic therapy in pig trachea: a fluence-response pilot study comparing Photofrin and mTHPC

**DOI:** 10.1038/sj.bjc.6690418

**Published:** 1999-05

**Authors:** L H P Murrer, K M Hebeda, J P A Marijnissen, W M Star

**Affiliations:** Department of Clinical Physics, Dr Daniel Den Hoed Cancer Centre, PO Box 5201, Rotterdam, 3008 AE, The Netherlands; Department of Pathology, St Radboud University Hospital Nijmegen, The Netherlands

**Keywords:** photodynamic therapy, light dosimetry, bronchus, Photofrin, mTHPC, normal tissue damage

## Abstract

The damage to normal pig bronchial mucosa caused by photodynamic therapy (PDT) using mTHPC and Photofrin as photosensitizers was evaluated. An endobronchial applicator was used to deliver the light with a linear diffuser and to measure the light fluence in situ. The applied fluences were varied, based on existing clinical protocols. A fluence finding experiment with short-term (1–2 days) response as an end point showed considerable damage to the mucosa with the use of Photofrin (fluences 50–275 J cm^−2^, drug dose 2 mg kg^−1^) with oedema and blood vessel damage as most important features. In the short-term mTHPC experiment the damage found was slight (fluences 12.5–50 J cm^−2^, drug dose 0.15 mg kg^−1^). For both sensitizers, atrophy and acute inflammation of the epithelium and the submucosal glands was observed. The damage was confined to the mucosa and submucosa leaving the cartilage intact. A long-term response experiment showed that fluences of 50 J cm^−2^ for mTHPC and 65 J cm^−2^ for Photofrin-treated animals caused damage that recovered within 14 days, with sporadic slight fibrosis and occasional inflammation of the submucosal glands. Limited data on the pharmacokinetics of mTHPC show that drug levels in the trachea are similar at 6 and 20 days post injection, indicating a broad time window for treatment. The importance of in situ light dosimetry was stressed by the inter-animal variations in fluence rate for comparable illumination conditions. © 1999 Cancer Research Campaign


*British Journal of Cancer* (1999) **80**(5/6), 744–755
© 1999 Cancer Research Campaign
Article no. bjoc.1998.0418

### Short- and long-term normal tissue damage with photodynamic therapy in pig trachea: a fluence-response pilot study comparing Photofrin and mTHPC

**LHP Murrer, KM Hebeda[2], JPA Marijnissen and WM Star[1]**

[1]Department of Clinical Physics, Dr Daniel Den Hoed Cancer Centre, University Hospital Rotterdam, PO Box 5201, 3008 AE Rotterdam, The Netherlands; [2]Department of Pathology, St Radboud University Hospital Nijmegen, The Netherlands

**Summary** The damage to normal pig bronchial mucosa caused by photodynamic therapy (PDT) using mTHPC and Photofrin as photosensitizers was evaluated. An endobronchial applicator was used to deliver the light with a linear diffuser and to measure the light fluence in situ. The applied fluences were varied, based on existing clinical protocols. A fluence finding experiment with short-term (1–2 days) response as an end point showed considerable damage to the mucosa with the use of Photofrin (fluences 50–275 J cm$^{-2}$, drug dose 2 mg kg$^{-1}$) with oedema and blood vessel damage as most important features. In the short-term mTHPC experiment the damage found was slight (fluences 12.5–50 J cm$^{-2}$, drug dose 0.15 mg kg$^{-1}$). For both sensitizers, atrophy and acute inflammation of the epithelium and the submucosal glands was observed. The damage was confined to the mucosa and submucosa leaving the cartilage intact. A long-term response experiment showed that fluences of 50 J cm$^{-2}$ for mTHPC and 65 J cm$^{-2}$ for Photofrin-treated animals caused damage that recovered within 14 days, with sporadic slight fibrosis and occasional inflammation of the submucosal glands. Limited data on the pharmacokinetics of mTHPC show that drug levels in the trachea are similar at 6 and 20 days post injection, indicating a broad time window for treatment. The importance of in situ light dosimetry was stressed by the inter-animal variations in fluence rate for comparable illumination conditions.

**Keywords:** photodynamic therapy; light dosimetry; bronchus; Photofrin; mTHPC; normal tissue damage

Photodynamic therapy (PDT) for treatment of lung cancer has been investigated extensively with the use of Photofrin as a photosensitizer (McCaughan et al, 1988; Hayata et al, 1996; Lam, 1994) for palliation, as well as with curative intent for treatment of early-stage lung cancer. Cortese et al (1997) showed that PDT with haematoporphyrin derivative (HpD) can be an alternative to surgery. Currently, the use of meta-tetrahydroxyphenyl chlorin (mTHPC) as a photosensitizing agent is being evaluated experimentally and clinically for the treatment of cancer of the lungs, oesophagus and larynx (Abramson et al, 1994; Lofgren et al, 1994; Grosjean et al, 1996).

The common method of light delivery in this application of PDT is the use of a linear (or cylindrical) diffuser, which is suited to illuminate hollow, cylindrical organs such as the trachea or the bronchi. The light dosimetry of this treatment consists of a measurement of the total output of the linear diffuser only, and the applied 'light dose' is defined as the total amount of light energy emitted per unit of length of the diffuser (J cm$^{-1}$). Little is known about other important factors that determine the light fluence rate (and fluence) distribution in the tissue, which is the appropriate 'light dose' to evaluate PDT effects.

In previous work we showed that the in situ fluence rate resulting from illumination of the bronchi with identical diffusers and output can vary considerably between individuals (Murrer et al, 1997a). This is related to the inter-individual variations in optical properties of the bronchial mucosa. Similar variations in fluence rate caused by variations in optical properties (with red light of 630 nm) are found in the bladder (D'Hallewin et al, 1992) and in the skin (Hudson et al, 1994). In addition, the positioning of the diffuser in the lumen (Marijnissen et al, 1993; Murrer et al, 1995) and the output characteristics of the linear diffuser used (Murrer et al, 1996) have great influence on the fluence rate distribution in the tissue.

Because so many, sometimes unknown, parameters can influence the fluence rate in the mucosa, it is necessary to perform an in situ measurement of the fluence rate to quantify the applied fluence. An applicator which makes it possible to perform such a measurement together with a controlled way of light delivery has been developed in our group (Murrer et al, 1997a). With the help of this applicator, the present study seeks to find a relationship between the applied fluence (true 'light dose') and the short-term (1–2 days) damage found in the normal (healthy) tracheal mucosa resulting from PDT with Photofrin or mTHPC. Consequently, the long-term damage resulting from an applied fluence that results in potentially recoverable short-term damage is investigated to determine the tolerance of normal tissue to PDT.

### METHODS

### General protocol

The study was performed in two parts. In the first (acute) part the normal tissue damage of the bronchial mucosa caused by PDT in the trachea of healthy pigs was assessed visually as well as histologically 24 and 48 h after illumination. Either mTHPC or Photofrin was used. The photosensitizer dose was kept the same in

*Received 11 November 1997*
*Revised 16 June 1998*
*Accepted 25 June 1998*

*Correspondence to:* LHP Murrer

**Table 1** Summary of the treatment parameters and light dosimetry

| No. | Site | Sensitizer | Interval injection-treatment (days) | Sacrificed on day | Fluence rate (mW cm$^{-2}$) (± 15%) | Output applicator (mW), (± 5%) | Total fluence (J cm$^{-2}$), (± 15%) | Diameter trachea (cm), (± 5%) | β (± 20%) |
|---|---|---|---|---|---|---|---|---|---|
| **Acute** | | | | | | | | | |
| | dist | mTHPC | 4 | 1 | 400 | 796 | 50 | 1.0 | 2.6 |
| 1 | mid | mTHPC | 4 | 1 | 400 | 796 | 25 | 1.0 | 2.6 |
| | prox | mTHPC | 4 | 1 | 400 | 507 | 12.4 | 1.3 | 5.4 |
| | dist | mTHPC | 4 | 1 | 400 | 859 | 50 | 0.9 | 2.0 |
| 2 | mid | mTHPC | 4 | 1 | 400 | 979 | 25 | 1.0 | 2.1 |
| | prox | mTHPC | 4 | 1 | 400 | 919 | 12.7 | 1.0 | 2.1 |
| | dist | Photofrin | 2 | 1 | 270 | 600 | 274 | 1.0 | 2.1 |
| 3 | mid | Photofrin | 2 | 1 | 257 | 600 | 133 | 0.8 | 1.6 |
| | prox | Photofrin | 2 | 1 | 330 | 600 | 79 | 0.9 | 2.4 |
| | dist | mTHPC | 4 | 2 | 400 | 1350 | 51 | 1.2 | 1.8 |
| 4 | mid | mTHPC | 4 | 2 | 400 | 1480 | 25 | 1.0 | 1.3 |
| | prox | mTHPC | 4 | 2 | 400 | 1220 | 12.6 | 1.2 | 2.0 |
| | dist | mTHPC | 4 | 2 | 400 | 1474 | 50 | 1.2 | 1.6 |
| 5 | mid | mTHPC | 4 | 2 | 400 | 1288 | 25 | 1.2 | 1.8 |
| | prox | mTHPC | 4 | 2 | 400 | 1165 | 12.7 | 1.2 | 2.0 |
| | dist | Photofrin | 2 | 2 | 188 | 600 | 190 | 1.2 | 2.0 |
| 6 | mid | Photofrin | 2 | 2 | 178 | 600 | 90 | 1.0 | 1.4 |
| | prox | Photofrin | 2 | 2 | 195 | 600 | 50 | 0.9 | 1.4 |
| **Follow-up** | | | | | | | | | |
| 1 | | mTHPC | 4 | 14 | 400 | 584 | 50 | 1.2 | 4.1 |
| 2 | | mTHPC | 4 | 10[a] | 400 | 605 | 50 | 1.1 | 3.7 |
| 3 | | Photofrin | 2 | –[b] | – | – | – | – | – |
| 4 | | mTHPC | 4 | 14 | 400 | 468 | 50 | 1.1 | 4.7 |
| 5 | | mTHPC | 4 | 14 | 400 | 620 | 50 | 1.2 | 4.1 |
| 6 | | Photofrin | 2 | 14 | 375 | 450 | 65 | 1.0 | 3.9 |

mTHPC dose was 0.15 mg kg$^{-1}$ bodyweight, Photofrin dose 2 mg kg$^{-1}$ bodyweight. [a]Sacrificed earlier because of non-PDT skin damage. [b]Excluded from the experiment because of uncertainty of the light dose applied. β is the measured fluence rate divided by the incident fluence rate. For further explanation see text.

all experiments and the total light fluence was varied (with constant fluence rate) in three areas in the trachea of each pig. This part served as a fluence-finding experiment for the second part. In the second part, one single total light fluence was applied and the bronchial mucosa damage was followed visually during the 2 weeks follow-up period and histologically after termination of the experiment. The total light fluence applied in the second part is the fluence that showed potentially recoverable damage in the first part for each drug applied. The experimental protocol (no. 604-96-02) adhered to rules laid down in the Dutch Animal Experimentation Act and was approved by the Committee on Animal Research of the Erasmus University Rotterdam.

### Animals and anaesthesia

The animals used in this study were female pigs of a cross-breed of Danish Landrace and Yorkshire pigs (~ 30 kg). The animals were kept on a standard diet. The day before treatment, as well as the day before follow-up bronchoscopy, they were kept fasted. For all procedures the animals were sedated with ketamine (intramuscularly, 10 mg kg$^{-1}$) and intubated. They were ventilated artificially with a mixture of nitrous oxide:oxygen (1:2 v:v) and 1.5% isoflurane. During ventilation, anaesthesia was maintained with pancorium (intravenously (i.v.) 4 mg) and fentanyl (i.v., 0.1 mg). After the experimental procedures the animals were hand-ventilated until independent breathing was restored. During the treatment the artificial ventilation duration never exceeded 45 min, during drug delivery and follow-up bronchoscopies the duration was less then 15 min. During drug delivery the animals were ventilated with a mask only and received no additional i.v. anaesthesia. The animals were sacrificed with a bolus injection of pentobarbital (i.v.).

### Drug delivery

In both the acute and follow-up part six animals were used. In each part two animals received Photofrin™ and four animals received mTHPC™. The Photofrin was kindly provided by Quadra Logic Technologies (Vancouver, Canada) and the mTHPC was kindly provided by Scotia Quantanova (Guildford, Great Britain).

The Photofrin was dissolved in a 5% dextrose solution and administered i.v. by a slow-push injection 48 h before treatment according to the Lederle/QLT protocol (P 503/504) for endobronchial PDT. The applied dose was 2 mg per kg bodyweight. The mTHPC was dissolved in a mixture of ethanol, polyethylene glycol and water that was supplied with the drug, and injected i.v. by a slow-push injection 96 h before treatment. This drug delivery-illumination interval is also used by other groups for mTHPC–PDT (e.g. Grosjean et al, 1996). The applied dose was 0.15 mg per kg bodyweight. The animals were housed separately in subdued light conditions during the entire study.

### Light delivery and dosimetry

The light was delivered using an applicator that was specifically developed for endobronchial PDT. The applicator incorporates a linear diffuser for light delivery, a fixation system that ensures the central positioning of the diffuser in the trachea and an isotropic probe for in situ measurement of the fluence rate on the trachea wall (Murrer et al, 1997a). A schematic of the endobronchial applicator is presented in Figure 1.

The design has been improved with a 1.5 cm diffuser with isotropic instead of forward scattering properties (Murrer et al, 1997b) manufactured by Rare Earth Medical (West Yarmouth, MA, USA). The isotropy of the diffuser causes the maximum of the fluence rate to be located on the trachea wall opposite the middle of the diffuser. The isotropic probe measures the fluence rate at this maximum.

With the aid of a Monte Carlo model for the geometry of a linear diffuser in a trachea (Murrer et al, 1995) we estimated an overlap of less than 5% of the maximum fluence rate between separate fields when a diffuser of 1.5 cm was used with an inter-lesion spacing of 5 cm. This is possible in the pig's trachea which is generally longer than the 15 cm ($3 \times 5$ cm) needed for the experiment.

The diameter of the isotropic probe fibre core has been increased by a factor of two compared to our first design, which causes a higher light output of the isotropic probe, enabling a more sensitive measurement. The isotropic probe (bulb diameter 0.8 mm) mounted on a 200 μm quartz core fibre was produced by Rare Earth Medical (West Yarmouth, MA, USA).

The light source used was a KTP/Nd:YAG laser with a dye unit (KTP/YAG 832 and Dye module series 600; Laserscope, San Jose, CA, USA) that was tuned to a wavelength of 652 or 630 nm for mTHPC or Photofrin respectively. The output of the linear diffuser was measured with an integrating sphere. The applicator was inserted in the sphere, unfolded and the laser was switched on. The voltage reading of a built-in photodiode was converted to the total output in mW with appropriate calibration factors for the two wavelengths used. The same integrating sphere was used to calibrate the isotropic probe in air (van Staveren et al, 1995). Built-in laser diodes provided a stable diffuse light field, which was calibrated with the same photodiode that was used for the output measurement. The output voltage of the photodiode was multiplied by an appropriate calibration factor to calculate the diffuse light field in the sphere for both wavelengths used.

When in contact with tissue, the reading of the isotropic probe calibrated in air has to be corrected because of the difference of refractive index ($n$) between air ($n = 1$) and tissue ($n = 1.37$). When the probe is pressed against the mucosa, the reading of the probe calibrated in air has to be multiplied by a factor 1.07 (± 2%) (Murrer et al, 1995, Marijnissen and Star, 1996). It is important that the isotropic probe is kept free of mucus and blood to ensure correct calibration. This can be checked through the bronchoscope and, if necessary, the probe must be rinsed through the bronchoscope or the applicator must be retracted, cleaned and inserted again (Murrer et al, 1997*a*). The light output of the isotropic probe was coupled to a photodiode inside a home-built dosimetry device, which displays the fluence rate as well as the integrated fluence rate in time (total fluence). The dosimeter is calibrated by inserting and unfolding the applicator in the sphere with the laser diodes switched on.

### Applied fluences

The fluence rates and the total fluences that were applied for the different sensitizers in the acute experiment were derived from either an existing protocol (Photofrin) or from protocols for, e.g. skin or ENT application (mTHPC) in humans.

The mTHPC-treatment fluence and fluence rate was derived from the incident fluences and fluence rates in protocols for, e.g. skin, where an incident fluence rate of 100 mW cm$^{-2}$ is used to a total incident fluence of 10 J cm$^{-2}$. In a pilot experiment on rats, we observed that the incident fluence rate on a shaved spot of skin (2 cm diameter) increased to 250% by backscattering. This factor serves as an approximation of the translation between the measurement of the incident fluence (irradiance) with a flat detector and the actual fluence rate measured with an isotropic probe touching the skin. Therefore we decided to take 2.5 times 10 J cm$^{-2}$ (flat detector) = 25 J cm$^{-2}$ (isotropic probe) as the central fluence and half and twice that fluence (12.5 and 50 J cm$^{-2}$) for the other two illumination fields. The highest total fluence was used on the field most distal to the animal's head, and the fluence decreased with more proximal location, thereby ensuring minimal relative overlap between illumination fields.

The increase of fluence rate (or fluence) above the incident irradiance (or incident fluence) at the surface of the mucosa appears to violate conservation of energy, but this is not the case. The fluence is a measure for the amount of energy *available* for absorption at a given point in the tissue. The actual amount of energy deposited in a volume element of the tissue is given by the fluence times the local absorption coefficient. Conservation of energy requires that the summation of the product of fluence and absorption coefficient over the entire volume equals the amount of energy in the incident beam minus losses in the form of reflection from and transmission through the medium.

Figure 2 shows the fluence rate in the tissue as a function of the distance from the surface. The (infinitely wide) surface is illuminated with a wide, collimated beam of light. The absorption and scattering properties used for the calculations are typical for bronchial mucosa at a wavelength of 630 nm (Murrer et al, 1995). In one case (solid line), the scattering by the tissue has been 'switched off', and the fluence rate decreases exponentially from the level of the incident irradiance (1 in the graph) at the tissue surface.

The second graph (dashed line) clearly shows the influence of scattering. The light scattering by the tissue causes the photons to deviate from their straight paths in the tissue, causing more light to be accumulated at relatively lower depths. As a result the fluence rate near the surface is increased 5–6 times compared to the incident irradiance (this factor depends on the exact optical properties of the tissue). Deeper in the tissue the fluence rate for the case with scattering drops below the fluence rate for the case without scattering. In short: increased scattering leads to higher fluence rates near the surface and shallower penetration of the light. The area under the curve for both sets of optical properties multipied by the absorption coefficients is a measure for the energy deposited in the tissue.

For superficial tumours (< 1 mm thickness) the fluence rate is nearly constant over the lesion volume, unless the lesion has an extremely (» 1 cm$^{-1}$) high absorption coefficient in the case of highly pigmented (tumour) tissue. The biological effect of PDT is then determined by the product of fluence and absorption coefficient of the photosensitizer (neglecting fluence rate effects). Surface light dosimetry is adequate in this case. Consider the optical properties of Figure 2. If the absorption coefficient of the photosensitizer is the same in both cases, the same biological effect requires a 5.7-fold smaller incident fluence in the case with light scattering, compared to zero light scattering, to achieve the same total fluence. For non-superficial tumours, the fluence at the

© Cancer Research Campaign 1999

**Table 2** Visual damage score for acute and follow-up experiment

| Acute | | | | | | | | | | | | | | | | | | |
|---|---|---|---|---|---|---|---|---|---|---|---|---|---|---|---|---|---|---|
| Sacrificed after | | | | | 1 day | | | | | | | | | | 2 days | | | |
| Animal | | 1 | | | 2 | | | 3 | | | 4 | | | 5 | | | 6 | |
| Sensitizer | | mTHPC | | | mTHPC | | | Photofrin | | | mTHPC | | | mTHPC | | | Photofrin | |
| Site | D | M | P | D | M | P | D | M | P | D | M | P | D | M | P | D | M | P |
| Redness | 0 | 0 | 0 | 0 | 0 | 0 | 2 | 2 | 1 | 0 | 0 | 0 | 0 | 0 | 0 | 2 | 2 | 1 |
| Whiteness | 0 | 0 | 0 | 0 | 0 | 0 | 0 | 0 | 0 | 1 | 1 | 1 | 1 | 1 | 1 | 0 | 0 | 0 |
| Oedema | 0 | 0 | 0 | 0 | 0 | 0 | 2 | 2 | 1 | 0 | 0 | 0 | 0 | 0 | 0 | 2 | 2 | 1 |
| Mucus | 1 | 1 | 1 | 0 | 0 | 0 | 1 | 1 | 1 | 0 | 0 | 0 | 0 | 0 | 0 | 1 | 1 | 1 |

| Follow-up | | | | | | | | | | | | | | | |
|---|---|---|---|---|---|---|---|---|---|---|---|---|---|---|---|
| Animal | | | 1 | | | | | 2[a] | | | | | 3[b] | | |
| Sensitiser | | | mTHPC | | | | | mTHPC | | | | | Photofrin | | |
| Day | 1 | 3 | 7 | 10 | 14 | 1 | 3 | 7 | 10 | 14 | 1 | 3 | 7 | 10 | 14 |
| Redness | 0 | 0 | 0 | 0 | 0 | 0 | 1 | 0 | 0 | – | – | – | – | – | – |
| Whiteness | 0 | 0 | 0 | 1 | 0 | 0 | 0 | 0 | 0 | – | – | – | – | – | – |
| Oedema | 0 | 0 | 0 | 0 | 0 | 0 | 0 | 0 | 0 | – | – | – | – | – | – |
| Mucus | 0 | 0 | 1 | 1 | 0 | 0 | 0 | 1 | 0 | – | – | – | – | – | – |
| Animal | | | 4 | | | | | 5 | | | | | 6 | | |
| Sensitizer | | | mTHPC | | | | | mTHPC | | | | | Photofrin | | |
| Day | 1 | 3 | 7 | 10 | 14 | 1 | 3 | 7 | 10 | 14 | 1 | 3 | 7 | 10 | 14 |
| Redness | 1 | 0 | 0 | 0 | 0 | 0 | 0 | 0 | 1 | 0 | 0 | 1 | 0 | 0 | 0 |
| Whiteness | 0 | 0 | 0 | 0 | 0 | 0 | 0 | 0 | 0 | 0 | 0 | 0 | 0 | 0 | 0 |
| Oedema | 0 | 1 | 0 | 0 | 0 | 0 | 1 | 0 | 1 | 0 | 0 | 0 | 1 | 0 | 0 |
| Mucus | 0 | 1 | 0 | 0 | 0 | 0 | 1 | 0 | 0 | 0 | 0 | 1 | 0 | 0 | 1 |

0 = none/normal, 1 = some/slight increase, 2 = much, – = not available. D = distal, M = middle, P = proximal. [a]Sacrificed on day 10 because of (non-PDT) skin damage. [b]Excluded from experiment because of unreliable dosimetry.

deepest boundary with normal tissue should be considered. If the lesion thickness is about 6.6 mm (optical properties of Figure 2), the fluence rate at this depth is the same, if the incident fluence is the same for both sets of optical properties. If the absorption coefficient of the photosensitizer is the same in both cases, the light fluence to be delivered at 6.6 mm depth is the same, so that the incident surface fluence to be applied in the case with light scattering should be the same as for the non-scattering case. If the tumour thickness is larger than 6.6 mm, all optical properties remaining the same, the incident fluence in the case with light scattering should be larger than without scattering, to achieve the same PDT effect at the deepest tumour–normal tissue boundary. For non-superficial lesions then, one needs a technique to measure the tumour thickness and a measurement of the light fluence at the appropriate depth.

To rule out fluence rate effects, the fluence rate was kept constant and the illumination time was varied to achieve different total fluences. The fluence rate was set at 400 mW $cm^{-2}$ as measured on the isotropic probe because this is the fluence rate we expected to find in the Photofrin treatment, based on ex vivo measurements (Murrer et al, 1995). Using the 2.5 times build-up factor described above, this fluence rate is equivalent to an incident collimated irradiance of about 160 mW $cm^{-2}$, comparable to incident fluence rates commonly used in mTHPC and Photofrin protocols. Treatment times were 62.5, 125 and 250 s for the three fields.

The protocol for Photofrin (QLT/Lederle P 503/504) prescribes a light output of 400 mW per cm diffuser length used for a duration of 500 s, yielding a total of 200 J per cm diffuser length. In the acute experiment we applied this protocol in one of the three fields illuminated. The other two fields were illuminated with half and twice this energy viz. 100 and 400 J per cm diffuser length with the same output in mW of the diffuser. The variation in energy applied was achieved by varying the time of exposure, leading to treatment times of 250, 500 and 1000 s.

Again the field with the highest total fluence was chosen most distally to the head of the animal and the resulting fluence rate and total fluence on the probe were recorded. The measured total fluence was used to determine a total fluence for the follow-up part of the experiment. In the follow-up part, the fluence rate on the probe was set at ≈ 400 mW $cm^{-2}$ (the same as the mTHPC experiment) and then the illumination was continued until the desired total fluence was reached.

### Build-up factor

Because of the scattering properties of the illuminated tissue, the fluence rate resulting from the illumination with the diffuser will be higher than the incident fluence rate on the mucosa. For diffusers with good isotropic properties (as used in our applicator) the maximum incident fluence rate for central placement in the lumen $\varphi_0$ (mW $cm^{-2}$) can be calculated from (Murrer et al, 1995):

$$\varphi_0 = P/\pi LD \arctan (L/D) \qquad (1)$$

with $P$ the total output power of the diffuser (mW), $L$ the length of the diffuser (cm) and $D$ the diameter of the trachea (cm). The build-up factor β is defined as the ratio of the measured fluence rate $\varphi_{true}$ and the incident fluence rate,

$$\beta = \varphi_{true}/\varphi_0 \qquad (2)$$

and depends on the optical properties of the mucosa.

### Treatment

The respiration tube (diameter 9 mm) was fixed just below the larynx to leave a maximum length of trachea exposed to be used

© Cancer Research Campaign 1999 

**Table 3** Histological damage scores for acute and follow-up experiment

| Sensitizer | mTHPC | | | | | | | | Photofrin | | | | | | | |
|---|---|---|---|---|---|---|---|---|---|---|---|---|---|---|---|---|
| **Sacrificed after (days)** | **1** | | | **2** | | | **14** | | **1** | | | **2** | | | **14** | |
| **Total fluence (J cm$^{-2}$)** | **50** | **25** | **12.5** | **50** | **25** | **12.5** | **50** | **c** | **274** | **133** | **79** | **190** | **90** | **50** | **65** | **c** |
| **Epithelium** | | | | | | | | | | | | | | | | |
| Reduction of mucus-producing cells | 0/1 | 1/1 | 1/2 | 2/0 | 2/1 | 2/2 | 0/1/1/1 | 0/0/0/0 | 2 | 2 | 2 | 2 | 2 | 2 | 1 | 0 |
| Acute inflammation | 0/0 | 0/0 | 0/1 | 1/0 | 1/1 | 1/1 | 2/0/1/1 | 1/0/0/1 | 1 | 1 | 2 | 0 | 1 | 1 | 0 | 0 |
| **Submucosa** | | | | | | | | | | | | | | | | |
| Acute inflammation | 1/1 | 1/2 | 2/2 | 1/0 | 1/1 | 1/1 | 1/1/1/1 | 0/0/0/0 | 1 | 1 | 2 | 2 | 2 | 2 | 0 | 0 |
| Oedema | 1/1 | 2/1 | 2/0 | 1/1 | 2/1 | 1/1 | 1/1/0/0 | 0/0/0/1 | 2 | 2 | 1 | 2 | 2 | 1 | 0 | 0 |
| Hyperaemia | 0/0 | 0/0 | 0/0 | 0/0 | 0/0 | 0/0 | 0/2/0/1 | 1/0/0/1 | 2 | 2 | 0 | 2 | 2 | 1 | 1 | 2 |
| Damage of blood vessels | 1/0 | 1/0 | 0/0 | 0/0 | 0/0 | 0/0 | 0/1/0/0 | 0/0/0/0 | 2 | 2 | 1 | 2 | 2 | 0 | 0 | 0 |
| Atrophy of submucosal glands | 1/0 | 1/1 | 0/2 | 1/1 | 2/1 | 2/1 | 0/2/0/1 | 0/0/0/0 | 2 | 2 | 2 | 1 | 1 | 2 | 1 | 1 |
| Acute inflammation of submucosal glands | 0/1 | 0/1 | 1/2 | 1/0 | 2/1 | 2/1 | 0/0/0/0 | 0/0/0/0 | 1 | 0 | 2 | 1 | 1 | 2 | 0 | 0 |
| Fibrosis | 0/0 | 0/0 | 0/0 | 0/0 | 0/0 | 0/0 | 0/1/0/1 | 0/0/0/0 | 0 | 0 | 0 | 0 | 0 | 0 | 1 | 0 |

No/none = 0, light/few = 1, severe/much = 2. Control values c are taken from sites in a treated trachea which received little or no light (distance from illuminated area ≥ 10 cm).

for illumination. A fibre bronchoscope with a large working channel (diameter 2.8 mm, Fujinon Medical Holland) was introduced through the tube, and the applicator was fed through the working channel. During this procedure the artificial ventilation was maintained through the open space between the bronchoscope and the tube. After unfolding the applicator the positioning of the detector was checked through the bronchoscope to ensure good contact with the mucosa and to ensure that there was no blood or mucus on the probe. The relative position of the applicator in the trachea was measured by the markings on the bronchoscope relative to the teeth of the animal. The larynx served as a reference for the zero position.

The applicator was fixed in the trachea such that the most proximal position was not too close to the tube to avoid reflections off the tube. In the case of the acute Photofrin treatment, the output of the diffuser was preset to a fixed output of 600 mW defined by the Photofrin protocol (1.5 cm diffuser × 400 mW cm$^{-1}$) and the time of exposure was measured. The resulting fluence rate and total fluence were recorded. In the other cases the laser was switched on and the output was adjusted such that the probe reading was 400 mW cm$^{-2}$, and kept at that level by adjusting the output if necessary. The treatment was halted when the desired total fluence was achieved. After the treatment the output of the applicator was measured, and the calibration of the probe was checked.

In the acute experiment half of the animals (one Photofrin, two mTHPC) were sacrificed at 24 and the other half at 48 h. In the follow-up experiment the animals were sacrificed after 14 days. On days 1, 3, 7, 10 and 14 the animals' tracheas were examined by bronchoscopy. In the case of excessive damage, the experiment was to be terminated earlier.

### Precautions

Several precautions were taken to avoid damage related to non-treatment light. During transport to and from the operating theatre the animals were shielded from light with dark cloth. In the operating theatre only necessary lights were left on, and those were not directed at the animals. The animals were also covered with dark cloth in theatre. The bronchoscope's light source was operated on the lowest setting allowing good visual control of the situation. During treatment the light source was switched off entirely. The duration of bronchoscopy with the light source on was kept to a minimum (1–2 min). During recovery after the experiments the animals were kept warm by cloth instead of using an infrared lamp.

### Tissue damage assessment

The damage to the normal mucosa was inspected both visually and histologically. The appearance of the mucosa was either photographed or recorded on videotape in the live animal. Images of the mucosa before treatment were made to serve as a reference. In the acute experiment, images were made after 24 or 48 h. Images were taken during each bronchoscopy in the follow-up experiment. The damage was specified qualitatively as an impression of the whole treatment field by specifying the amount of red and white discolouration, oedema and increase of mucus formation on the mucosa. The damage was scored according to: none/normal = 0, some/slightly increased = 1, much = 2.

After the experiment, the tracheas were excised and fixed in buffered formaldehyde (4%). After fixation, the material was cut and haematoxylin and eosin (H&E) stained for histological analysis. Longitudinal strips of the whole trachea as well as rings from the centre of the treated areas were examined. The histological effects of the treatment were judged partly qualitatively (for example the nature of the inflammatory cells) and partly quantitatively (for example the number of inflammatory cells and the number of mucus producing cells in the epithelium). The damage was scored for a panel of histological characteristics according to no/none = 0, light/few = 1, severe/much = 2. Of these, the most relevant are discussed in the results.

*© Cancer Research Campaign 1999*

### mTHPC tissue level determination

Tissue samples were taken from the mTHPC-treated animals after they were sacrificed at 6 and 20 days after injection (2 and 14 days after treatment). Samples were taken from the skin, trachea (cartilage and mucosa in one sample) and oesophagus and were snap-frozen in liquid nitrogen for further processing. The method of determining the mTHPC tissue levels with high-performance liquid chromatography (HPLC) is described by Wang et al (1993).

### RESULTS

In Table 1 an overview is given of the treatment parameters. The treatment sites are coded dist (distal), mid (middle) and prox (proximal) indicating their position relative to the larynx of the animal. For each animal the output of the applicator's diffuser is specified as well as the measured fluence rate and the total fluence as measured by the isotropic probe.

### General comments

The treatment and follow-up in the acute part of the experiment was conducted as planned without problems. In the follow-up experiment one animal (no. 3) was excluded from the study because of technical problems during the illumination that led to uncertainty about the actual light dose delivered. Furthermore, animal 2 from the follow-up group had to be sacrificed earlier (day 10) because of damage to the skin of the belly of the animal. To the best of our knowledge, this damage was not PDT-related because three other animals with the same drug dose that were housed and treated under exactly the same conditions showed no signs of this skin damage. Moreover, the mTHPC tissue levels of damaged and undamaged skin samples were not excessively high (10–50 ng $g^{-1}$, see section on mTHPC levels). Probably the animal was accidentally exposed to concentrated, alkaline detergent. Except for this incident, the general condition of all animals was good during the experiments.

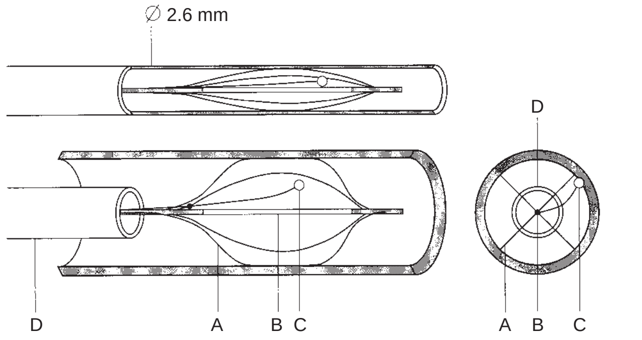

**Figure 1** Applicator for light delivery and light dosimetry in the bronchial tree. (Top) Schematic drawing of the folded applicator in the catheter and (bottom) of the applicator unfolded in a cylindrical lumen. The cross-section shows the position of the detector bulb that is pressed against the wall. The separate components are the light-diffusing fibre at the centre (B), the isotropic detector (C) and the steel springs of the fixation basket (A). The outer diameter of the nylon tubing (D) that contains the folded applicator is 2.6 mm. The black dot shown near the proximal end of the applicator indicates where the detector fibre is attached to the steel spring

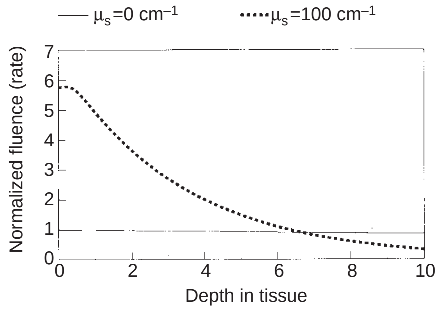

**Figure 2** Fluence (rate) in tissue for an infinitely wide light beam incident on a flat, semi-infinite medium of constant optical properties. The fluence (rate) is normalized to the incident irradiance. Both curves: the absorption coefficient $\mu_a$ = 0.15 $cm^{-1}$, the anisotropy factor $g$ = 0.8 and the index of refraction of the medium $n$ = 1.37. Solid line: $\mu_s$ = 0 $cm^{-1}$. Dashed line: $\mu_s$ = 100 $cm^{-1}$. The curves were calculated with Lambert-Beer's law ($\mu_s$ = 0 $cm^{-1}$) and the δ-E(4) approximation ($\mu_s$ = 100 $cm^{-1}$; Star, 1995)

### DOSIMETRY

In the case of the acute Photofrin experiment the diffuser output was fixed and the fluence rate was measured and found to be stable within 5%, indicating good fixation of the diffuser and the isotropic probe. The output of the diffuser was checked afterwards and was found to be stable within 5%.

In the other situations where the output was varied to achieve a fluence rate of ~ 400 mW $cm^{-2}$ the output of the diffuser could be adjusted within seconds after the start of the illumination to reach the desired level. Once this level was reached, only minor adjustments of the output (≤ 5%) were needed to have a stable (within 5%) reading on the isotropic probe.

The diameters of the tracheas were measured in the microscopic slides and corrected for the 10% shrinkage due to fixation (Burck, 1973). The diameters were used to calculate the incident fluence rate and the build-up factor for every field illuminated. The average trachea diameter in the acute experiment was 1.06 (standard deviation (s.d.) 0.14) cm, and in the follow-up experiment the average diameter was 1.12 (s.d. 0.08) cm, which is not significantly different despite the 12- or 13-day age difference between the two groups of animals.

There was no clear difference in β values between 630 (β = 2.1; s.d. 0.9) and 652 nm (β = 2.6; s.d. 1.2). There was a pronounced difference, however, in β values between the acute (β = 2.1; s.d. 0.9) and the follow-up group (β = 4.1; s.d. 0.4), which also existed for the two separate wavelengths. The overall average β value found was 2.5 (s.d. 1.2) for all animals and wavelengths.

### Visual damage score

During each bronchoscopy, an image of the treated areas was taken and the damage was described qualitatively by specifying the amount of red and white discolouration, oedema and increase of mucus formation on the mucosa. The scores indicated in Table 2 give an indication of the entire field treated.

In the acute experiment, the most pronounced damage is found in the animals treated with Photofrin (3 and 6). All three fields illuminated showed redness and oedema in both animals, with slightly less damage at the proximal site where the lowest fluence was applied. There was no clear difference between the damage observed after 24 and after 48 h. The mTHPC-treated animals (1, 2, 4 and 5) showed very little effect in all animals with all applied fluences. Some whitish discolouration of the tissue was found after 48 h (animals 4 and 5), which was not seen at 24 h. In animal 1 (after 24 h) some mucus was observed.

© Cancer Research Campaign 1999 

A

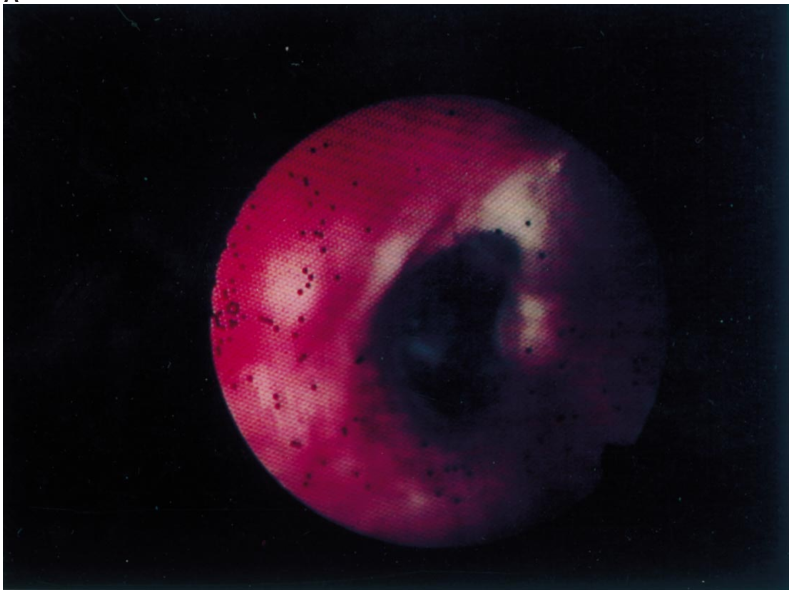

Photofrin

B

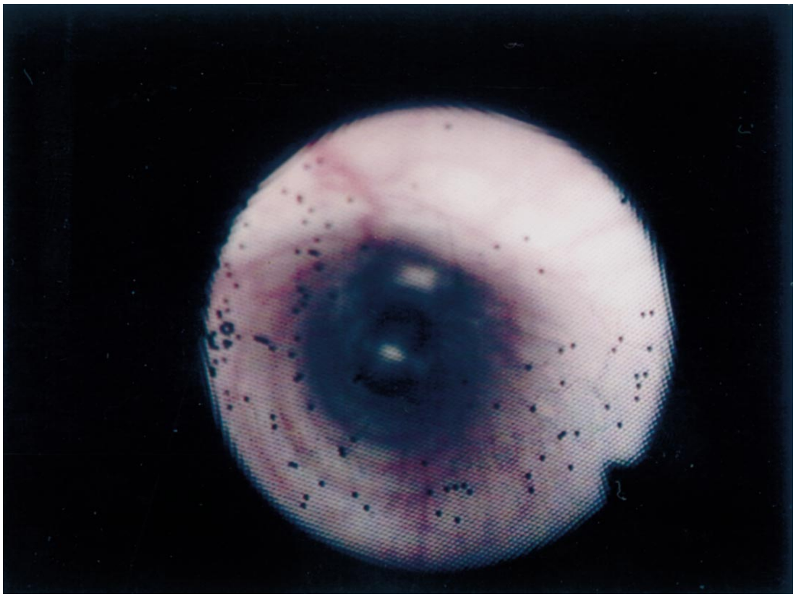

mTHPC

**Figure 3** Bronchoscope image of the part of the trachea illuminated with the highest fluence for both sensitizers taken 24 h after treatment. (**A**) Photofrin treatment with 274 J cm$^{-2}$ (630 nm). (**B**) mTHPC treatment with 50 J cm$^{-2}$ (652 nm). The black dots in both pictures are bronchoscope imaging artefacts

In Figure 3, bronchoscope images are shown of the areas treated with the highest fluences for both sensitizers, taken 24 h after treatment. In the image taken in the Photofrin-treated animal the red discolouration (haemorrhage, see Histology), swelling (oedema) and some excess mucus formation are clearly visible (fluence 274 J cm$^{-2}$). The image of the mTHPC-treated animal (fluence 50 J cm$^{-2}$) shows mucosa with the same appearance as before treatment.

An overview of the tracheas treated with three fluences and excised and fixed 48 h after treatment is presented in Figure 4. The Photofrin-treated trachea shows three bands of dark discolouration, indicating areas where haemorrhage has occurred. The width of the dark areas is (left to right) 2.5, 2 and 1.5 cm for fluences of 190, 90 and 50 J cm$^{-2}$ respectively. The trachea of the Photofrin-treated animal that was sacrificed after 24 h also showed dark discoloured areas for the middle (133 J cm$^{-2}$) and high (271 J cm$^{-2}$) fluences, although not as sharply demarcated as in the animal sacrificed after 48 h. No clear region of damage was observed for the lowest fluence (79 J cm$^{-2}$). The mTHPC-treated tracheas showed no macroscopical treatment effects, the appearance is quite comparable to that of a normal, untreated trachea (see Figure 4).

*© Cancer Research Campaign 1999*

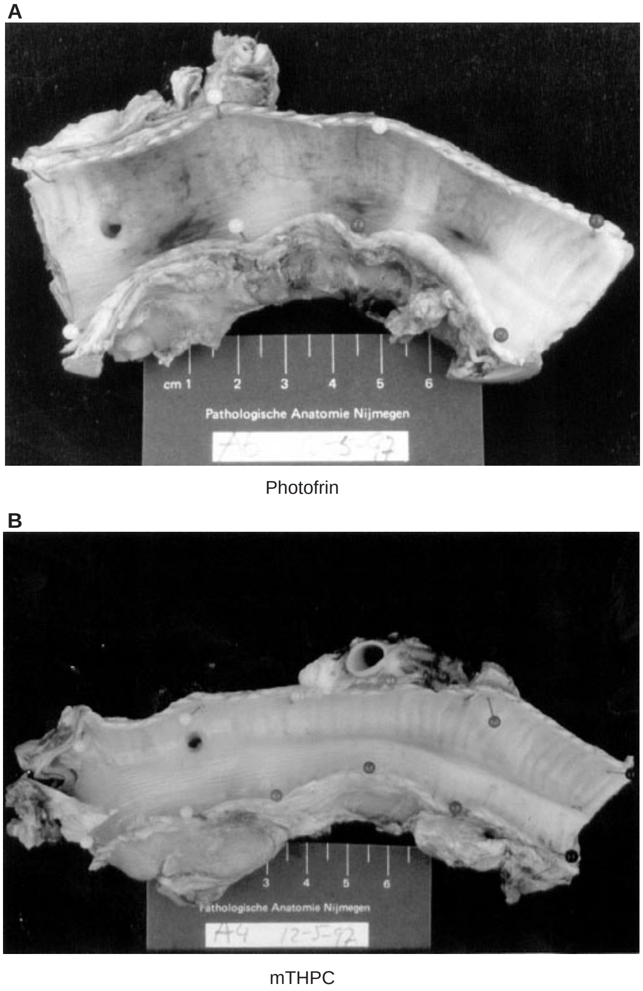

Figure 4 Overview picture of the dissected and fixed tracheas of animals sacrificed 48 h after treatment. The tracheas were cut lengthwise and opened. The larynx was located at the right side. (**A**). Photofrin treatment with (left to right) 190, 90 and 50 J cm$^{-2}$ (630 nm). (**B**). mTHPC treatment with (left to right) 50, 25 and 12.4 J cm$^{-2}$ (652 nm)

In the follow-up experiment the overall damage observed visually was mild (as intended). The mTHPC-treated animals showed some whitish discolouration and mucus on different days of the follow-up varying from day 3 to day 10. On day 14 the situation was normal again. The Photofrin-treated animal showed some redness on day 3 and some oedema on day 7 (probably non-specific because of the long time span after illumination). On day 14 the situation had returned to normal, apart from some mucus formation.

### Histology

The major changes in histology are summarized in Table 3, and some examples of the histology are shown in Figure 4. No systematic inter-animal variations were observed. A slight chronic inflammation was present directly beneath the epithelium (lamina propria) in all animals and was considered as non-specific. For all experiments the changes were limited to the trachea lining epithelium (lamina propria) and the submucosa. The cartilage or deeper tissues were not affected in any of the groups, except for fluence

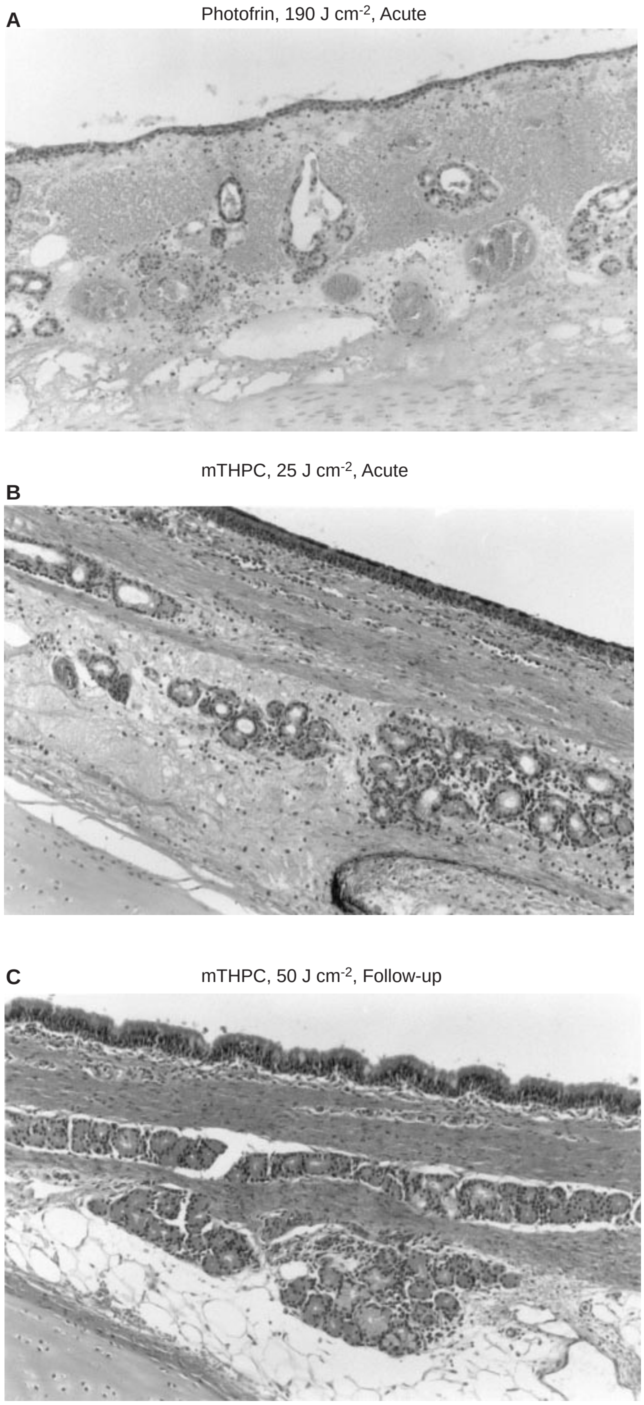

**Figure 5** Histological slides of the acute and the follow-up experiment (HE stained). (**A**) Damage to the trachea 48 h after illumination of a Photofrin-injected animal with a fluence of 190 J cm$^{-2}$. The epithelium is atrophic. The submucosal glands and blood vessels are damaged and surrounded by oedema. PMN cells are absent, only sporadic mononuclear cells are seen. (magnification 25×). (**B**). Damage 48 h after illumination (mTHPC, 25 J cm$^{-2}$). The epithelium shows loss of mucus producing cells. In the submucosa some oedema and a slight diffuse infiltration of mononuclear cells is seen. The glands show slight atrophic changes (magnification 100×).(**C**) Damage 2 weeks after illumination (mTHPC, 50 J cm$^{-2}$). The trachea appears normal, with epithelium containing mucus-producing cells, seromucinous glands without atrophy and only slight focal infiltration with mononuclear cells as is seen in the control tissues. Fibrosis is lacking (magnification 100×)

*© Cancer Research Campaign 1999*

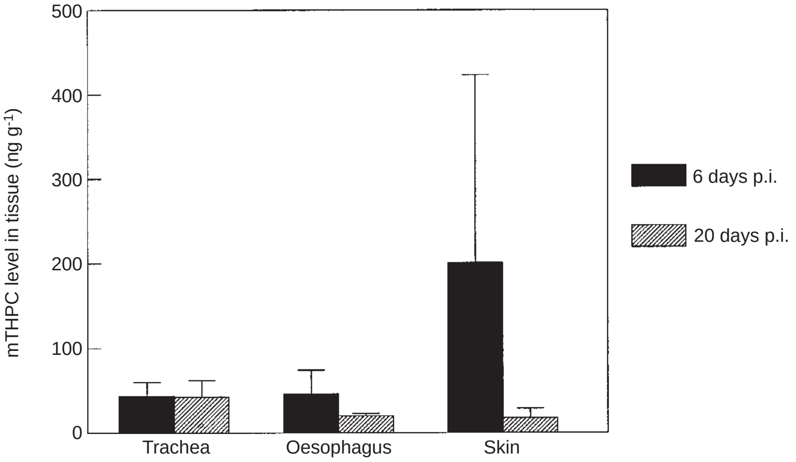

**Figure 6** mTHPC levels of pig tissues, taken from animals sacrificed 2 days (acute animals 4 and 5) and 14 days after treatment (follow-up animals 1, 4 and 5). Bars indicate the standard deviations independent circumscript mononuclear infiltrates that were found in the oesophageal glands in two of three mTHPC- and zero of one Photofrin-treated animals in the acute experiment (of one mTHPC and one Photofrin animal, no oesophageal tissue was available in the acute part). In the long-term study oesophageal glands of zero of four mTHPC and two of two Photofrin animals were found to contain these infiltrates.

Changes of the respiratory epithelium of the trachea consisted of increasing seriousness of loss of mucus producing cells, diminishing number of nuclear rows, flattening of the cells, loss of cilia and denudation and influx of polymorphonuclear (PMN) cells. The seromucinous submucosal glands lining the trachea were also involved in therapy-related damage. They showed an increase of infiltrating mononuclear cells and, immediately after treatment, of PMN cells. Loss of mainly mucinous cells, flattening of the epithelial cells and widening of acini is referred to as atrophy of submucosal glands. Mainly, the deep submucosal blood vessels (near the cartilage) showed hyperaemia. Damage of blood vessels in the lamina propria and submucosa consisted of frank destruction of endothelium (nuclear debris and disappearance of endothelium without inflammatory reaction) and stasis. Only very small areas with accumulation of coarse collagen bundles were seen occasionally near the submucosa at 2 weeks after illumination.

### *mTHPC*

In the dose-finding experiment no clear correlation between the applied fluence and severity of changes was observed in the mTHPC group. A quite intense inflammatory reaction with oedema and infiltrating PMN cells was seen in the lamina propria and submucosa for all fluences at day 1 after treatment, while the epithelium showed a slight or focal decrease of mucus-producing cells without signs of acute inflammation. In one animal the submucosal blood vessels showed very slight damage. At 2 days after treatment, inflammation and oedema were less while mucus producing cells were almost lacking in the epithelium.

In the follow-up experiment the changes in the treated part of the trachea compared to the untreated (Table 3) part were only slight and could be found only in some animals. The changes consisted only of minor loss of mucus-producing cells in the epithelium and submucosal glands combined with slight acute inflammation with some oedema of the submucosa and some hyperaemia. Small areas of fibrosis were found in the submucosa of two of four animals.

### *Photofrin*

All treated areas in the Photofrin-treated animals showed an immediate severe denudation of the epithelium and loss of mucus-producing cells that persisted during the second day of treatment. At both 1 and 2 days after treatment the high fluences caused severe vascular damage in the lamina propria and submucosa with stasis, fragmentation of endothelial nuclei, karyolysis and severe oedema. These severe vascular changes were not observed in mTHPC-treated animals. One day after treatment a surprisingly slight infiltration of PMN cells was seen in the Photofrin-treated animals. Only at day 2 the acute inflammation was severe in all irradiated fields. Two weeks after the treatment with 65 J $cm^{-2}$ little difference was seen between the irradiated spot and the control area. (The control spot of the Photofrin-treated animal that was excluded from the experiment showed the same characteristics.) A slight loss of submucosal glands and a small area of fibrosis were found in the submucosa of the treated spot.

### Applied fluences for the follow-up experiment

The fluences used in the follow-up experiment were 50 J $cm^{-2}$ and 65 J $cm^{-2}$ for mTHPC and Photofrin respectively. The fluences with potentially recoverable damage were chosen based on the effects observed in the acute experiment, with the observed visual and histological damage as a guideline.

For the mTHPC treatment the highest applied fluence of the acute experiment was used, because both visually and histologically no severe effects (such as blood vessel damage) were observed. The Photofrin fluence was chosen as an average of the lowest dose applied in both animals in the acute experiment. In this fluence range severe effects started to show as a dark band of haemorrhage with 79 J $cm^{-2}$ in the first and no such area in the second animal with a fluence of 50 J $cm^{-2}$. Histologically, these dark bands are the result of hyperaemia, severe damage to blood vessels and oedema.

 *© Cancer Research Campaign 1999*

### mTHPC tissue levels

The levels of mTHPC were determined in trachea, oesophagus and belly skin samples, the data are shown in Figure 4. The trachea sample (ring height: 5 mm) was taken from a distal part that received little or no light, and included cartilage as well as mucosa. The oesophagus sample was taken from the same region (section of 5 mm). The average value for the mTHPC level in the trachea is 44 ng $g^{-1}$ ($n$ = 2) at 6 days and remains at a level of 46 ng $g^{-1}$ ($n$ = 3) at 20 days after injection. The oesophagus level decreases from 46 ng $g^{-1}$ ($n$ = 2) at 6 days to 20 ng $g^{-1}$ ($n$ = 3) at 20 days. The skin drug level at 6 days was highly variable with a value of 358 and 44 ng $g^{-1}$ for two different animals. The level at 14 days is more consistent at 18 ng $g^{-1}$ ($n$ = 3), comparable to the oesophagus level.

### DISCUSSION

### Normal tissue damage with Photofrin and mTHPC

During the acute experiment we observed a distinct difference in normal tissue damage between the Photofrin and mTHPC treatment. The fluences used were based on values used for treatment of tumours in clinical practice where excessive normal tissue damage is to be avoided. Therefore such a distinct difference was not expected. It must be noted that the fluence used in the clinical QLT/Lederle Photofrin protocol was poorly defined because this protocol only specifies the output of the linear diffuser irrespective of the lumen diameter and the optical properties of the mucosa. The mTHPC total fluences chosen in this study were comparable to those employed by Savary et al (1994), who used fluences up to 16 J $cm^{-2}$ of *incident* light, roughly comparable with the 50 J $cm^{-2}$ *total* fluence we used (with the conversion factor 2.5, see Methods). Photofrin protocols for the treatment of skin lesions use total irradiances (*incident* fluences) of 50–100 J $cm^{-2}$ or higher (Wilson et al, 1992) with acceptable normal tissue response. With the conversion factor of 2.5 this indicates a fluence range of 125–250 J $cm^{-2}$. These fluences are somewhat higher than the total fluences we measured for the illuminated fields treated according to the QLT/Lederle protocol, viz. 133 and 90 J $cm^{-2}$ (mid sections in the acute animals 3 and 6). In these fields considerable normal tissue damage was observed at fluences that induce no severe side-effects in the treatment of skin lesions. Obviously, the light dosimetric conversion is just one aspect to take into account when adapting a protocol written for a specific target for use in an other organ.

In the mTHPC fluence-finding experiment no clear correlation between the applied fluence and the normal tissue damage was observed. The fluence range that was investigated might be below threshold for the induction of significant effects. Abramson et al (1994) observed an illumination threshold for the induction of oedema and erythema in the canine larynx. Unfortunately, the absolute fluence levels of Abramson's study and the present work cannot be compared because the former only specifies the diffuser output.

The fluences applied in the follow-up experiment did not induce severe long-term effects (14 days) as could be observed visually as well as histologically. This result suggests that, while treating a lesion in the trachea, the normal (sub)mucosa of the trachea of the pig can withstand a fluence of ~ 50 J $cm^{-2}$ for both sensitizers for the drug dose and interval between injection and treatment used here. The maximum tolerable fluence for mTHPC might be even higher, as we tested the highest fluence from the acute experiment in the follow-up experiment. Fifty Joules $cm^{-2}$ fluence can be compared (with the reservations noted above) to an incident irradiation of ~ 20 J $cm^{-2}$, which is already a relatively high 'light dose' for skin treatments.

One has to bear in mind, however, that the presence of (pre-) malignant tissue can alter the pharmacokinetics locally and alter the sensitivity of the surrounding tissue to PDT. The pharamacokinetics could be monitored in vivo by a fluorescence measurement to evaluate the influence of the presence of malignant tissue (Monnier et al, 1990; Braichotte et al, 1996). A pig tumour model in the trachea could give more insight in this matter, possibly the technique used by Hayata et al (1983) to induce an invasive squamous cell carcinoma in a canine model could be used in the pig also.

### Depth of damage

For both sensitizers and all fluences employed, the damage was limited to the mucosa and the submucosa and did not extend into or beyond the cartilage. The cartilage structure contains little blood and has therefore a low oxygen and photosensitizer content (Lofgren et al, 1994), and is relatively insensitive to photodynamic therapy. Lofgren observed a concentration of 10 ng $g^{-1}$ of mTHPC in the cartilage as opposed to 80 ng $g^{-1}$ in the mucosa of the canine larynx, 6 days after injection of 0.3 mg $kg^{-1}$ mTHPC.

There was no clear light demarcation between damaged and non-damaged zones in the mucosa for both sensitizers at all fluences applied. To look into this, the light penetration in the bronchial mucosa was estimated with the help of a Monte Carlo model for this geometry (Murrer et al, 1995, 1998). The optical properties of the mucosa were varied to cover a range of possible conditions during illumination. The scattering coefficient was varied from 50 to 100 $cm^{-1}$ ($g$ = 0.77) and the absorption was varied from 0.05 to 0.55 $cm^{-1}$. This range was set around the optical properties we reported earlier for bronchial mucosa (Murrer et al, 1995). We found that the penetration in the mucosa (which is about 1 mm thick) was comparable for all combinations of optical properties in the set. This was estimated by taking the average ratio of the fluence rate at 1 mm depth and at the mucosa surface, which yielded a value of 0.7 (s.d. 0.1). This indicates that there is no sharp drop in fluence rate to be expected in the 1 mm mucosa tissue which would cause a demarcation between damaged and non-damaged tissue. Apparently, the thickness of the cartilage region (3–4 mm) reduces the fluence rate sufficiently to prevent induction of observable damage outside the trachea.

### Vascular effects

Irreversible vascular damage was observed in the acute Photofrin experiment. This damage may explain the fact that the influx of PMN cells (acute inflammation) seems delayed for the highest fluence rates used. At day 1, only for the lowest fluence applied an extensive influx of PMN cells is seen, while for the areas treated with higher fluences severe inflammation only occurs at day 2 (Table 3).

Also with mTHPC vascular effects have been reported (Lofgren et al, 1994). In our study, vascular effects seem slight and reversible. Contrary to the Photofrin findings, PMN cell infiltration was at its maximum on day 1 and reduced on day 2. This suggests vascular spasm instead of destruction of the vasculature as seen in the Photofrin animals.

© Cancer Research Campaign 1999

Fluorescence imaging after injection of mTHPC shows that the drug content of vascular endothelium reaches a peak at 96 h and then decreases again (Andrejevic et al, 1996; Savary et al, 1997). However, Menezes da Silva et al (1995) showed that most extensive vascular occlusion after illumination occurred at 11 h after mTHPC injection, independently of the blood serum level (peak at 3 h). Their explanation is that vascular damage must result from damage to other cells than endothelial cells. Our illuminations were performed much later (96 h) than this time point of maximum vascular sensitivity to mTHPC–PDT, thus possibly explaining the lack of irreversible vascular damage in our study.

### mTHPC tissue levels

The level of mTHPC in the trachea was determined from samples containing both the mucosa and the cartilage. From the fact that the cartilage contains little mTHPC (Lofgren et al, 1994) it can be concluded that the levels in the mucosa only are higher than those indicated in Figure 4. The fact that the mTHPC level in the trachea stays at the same level between 6 and 20 days after injection indicates that the day of treatment can be chosen over a long period of time (assuming that the ratio of the cartilage/mucosa levels does not change).

### Dosimetry: the build-up factor β

The β-values found in the experiment are lower than the values found in previous ex vivo experiments (Murrer et al, 1995), and this can be attributed to the absence of blood in the ex vivo experiment. The blood in the mucosa in the in vivo situation increases the absorption of the treatment light, thereby lowering the build-up factor. The β-values found in this experiment agree with those reported in earlier in vivo experiments on pigs, while β-values in human volunteers are higher (average 4.7, s.d. 2.2, $n = 3$) (Murrer et al, 1997*a*).

The β-values show large inter-animal variations (average 2.5, s.d. 1.2), as was also observed in measurements in humans (Murrer et al, 1997*a*). This shows the necessity of in situ fluence rate measurements to assure equal illumination conditions. The presence of tumour tissue in the trachea may alter the dosimetry. Lesions often present as a region of whitish discolouration, representing areas with relatively low absorption of light, causing a locally higher build-up factor. Haemorrhages (or blood vessels located near the mucosa surface) induce locally increased absorption and consequently a lower build-up factor. These influences of local ‘colour’ are reflected in the intra-animal variations in β (Table 1).

The difference in β between the acute and the follow-up group (for both wavelengths) may be caused by variations in the oxygenation of the blood of the animals. The absorption of the light by the blood depends on the oxygenation of the blood (Cheong et al, 1990), which is influenced by the anaesthesia. The anaesthesia during the two parts of the experiments was conducted by different operators, which may explain the variations.

### ACKNOWLEDGEMENTS

The authors wish to thank Joos Heisterkamp, Enno Collij, Rob Meijer, Henk Bronk and Roy Spruyt at the Laboratory of Experimental Surgery as well as Otto Speelman, Dennis Francois and Ronald van Loon for support during the experiments. Scotia Quantanova and QLT are acknowledged for generously supplying the photosensitizers (mTHPC and Photofrin respectively). Jerry van der Ploeg and Edward Donkersloot are greatly acknowledged for the construction of the bronchus applicators. The histological sections were prepared at the department of pathology of the St Radboud University Hospital Nijmegen. The mTHPC tissue levels were determined at the lab of Dr Lim of the MRC Toxocology Unit, University of Leicester (UK). This work was supported by the Dutch Cancer Society, grant DDHK 93-615.

#### REFERENCES

Abramson AL, Lofgren LA, Ronn AM, Nouri M and Steinberg BM (1994) Treatment effects of meta-tetra-(hydroxyphebyl)chlorin on the larynx. In *New Approaches to Cancer Treatment: Unsaturated Lipids and Photodynamic Therapy,* Horrobin DF (ed), pp. 142–147. Churchill Communications Europe: London

Andrejevic S, Savary J-F, Monnier P, Fontolliet C, Braichotte D, Wagnières G and van den Bergh H (1996) Measurements by fluorescence spectroscopy of the time-dependent distribution of meso-tetra-hydroxyphenyl-chlorin in healthy tissues and chemically induced ‘early’ squamous cell carcinoma of the Syrian hamster cheek pouch. *Photochem Photobiol B* **36**: 143–151

Braichotte D, Savaray J-F, Glanzmann T, Monnier P, Wagnières G and van den Bergh H (1996) Optimizing light dosimetry in photodynamic therapy of the bronchi by fluorescence spectroscopy. *Lasers Med Sci* **11**: 247–254

Burck H-C (1973) *Histoligische Technick*, pp. 46–47. Georg Thieme Verlag: Stuttgart

Cheong WF, Prahl SA and Welch AJ (1990) A review of optical properties of biological tissues. *IEEE J Quantum Electron* **26**: 2166–2185

Cortese DA, Edell ES and Kinsey JH (1997) Photodynamic therapy for early stage squamous cell carcinoma of the lung. *Mayo Clin Proc* **72**: 595–602

D’Hallewin MA, Baert L, Marijnissen JPA and Star WM (1992) Whole bladder wall photodynamic therapy with in situ light dosimetry for carcinoma in situ of the bladder. *J Urol* **148**: 1152–1155

Grosjean P, Savary J-F, Wagnières G, Mizeret J, Woodtli A, Thuemann J-F, Fontolliet C, van den Bergh H and Monnier P (1996) Tetra(m-hydroxyphenyl)chlorin clinical photodynamic therapy of early bronchial and oesophageal cancer. *Lasers Med Sci* **11**: 227–235

Hayata Y, Kato H, Konaka C, Hayashi N, Tahara M, Saito T and Ono J (1983) Fiberoptic bronchoscopic photoradiation in experimentally induced canine lung cancer. *Cancer* **51**: 50–56

Hayata Y, Kato H, Furuse K, Kusunoki Y, Suzuki S and Mimura S (1996) Photodynamic therapy of 168 early stage cancers of the lung and oesophagus: a Japanese multi-center study. *Lasers Med Sci* **11**: 255–259

Hudson EJ, Stringer MR, Cairnduff F, Ash DV and Smith MA (1994) The optical properties of skin tumours measured during superficial photodynamic therapy. *Lasers Med Sci* **9**: 99–103

Lam S (1994) Photodynamic therapy of lung cancer. *Semin Oncol* **21**: 15–19

Lofgren LA, Ronn AM, Abramson AL, Shikowitz MJ, Nouri M, Lee CJ, Batti J and Steinberg BM (1994) Photodynamic therapy using m-tetra-(hydroxyphenyl)chlorin: an animal model. *Arch Otolaryngol Head Neck Surg* **120**: 1355–1362

McCaughan JS, Hawley PC, Bethel BH and Walker J (1988) Photodynamic therapy of endobronchial malignancies. *Cancer* **62**: 691–701

Marijnissen JPA and Star WM (1996) Calibration of isotropic light dosimetry detectors based on scattering bulbs in clear media. *Phys Med Biol* **41**: 1191–1208

Marijnissen JPA, Baas P, Beek JF, van Moll JH, van Zandwijk N and Star WM (1993) Pilot study on light dosimetry for endobronchial photodynamic therapy. *Photochem Photobiol* **58**: 92–98

Menezes da Silva FA and Newman EL (1995) Time-dependent photodynamic damage to blood vessels: correlation with serum photosensitizer levels. *Photochem Photobiol* **61**: 414–416

Monnier P, Savary M, Fontolliet C, Wagnières G, Chatelain A, Cornaz P, Depeursinge C and van den Bergh H (1990) Photodetection and photodynamic therapy of ‘early’ squamous cell carcinomas of the pharynx, the oesophagus and the tracheo-bronchial tree. *Lasers Med Sci* **5**: 149–168

Murrer LHP, Marijnissen JPA and Star WM (1995) Ex vivo light dosimetry and Monte Carlo simulations for endobronchial photodynamic therapy. *Phys Med Biol* **40**: 1807–1817

 *© Cancer Research Campaign 1999*

Murrer LHP, Marijnissen JPA and Star WM (1996) Light distribution by linear diffusing sources for photodynamic therapy. *Phys Med Biol* **41**: 951–961

Murrer LHP, Marijnissen JPA, Baas P, van Zandwijk N and Star WM (1997*a*) Applicator for light delivery and in situ light dosimetry during endobronchial photodynamic therapy: first measurements in humans. *Lasers Med Sci* **12**: 253–259

Murrer LHP, Marijnissen JPA and Star WM (1997*b*) Improvements in the design of linear diffusers for photodynamic therapy. *Phys Med Biol* **42**: 1461–1464

Murrer LHP, Marijnissen JPA and Star WM (1998) Monte Carlo simulations for endobronchial photodynamic therapy: the influence of variations in optical and geometrical properties and of realistic and eccentric light sources. *Lasers Surg Med* **22**: 193–206

Savary J-F, Monnier P, Wagnières G, Braichotte D, Fontolliet C and van den Bergh H (1994) Preliminary clinical studies of photodynamic therapy with mesa-tetrahydroxyphenyl chlorin (m-THPC) as a photosensitising agent for the treatment of early pharyngeal, oesophageal and bronchial carcinomas. *Proc Spie* **2078**: 330–340

Savary J-F, Monnier P, Fontolliet C, Mizeret J, Wagnières G, Braichotte D and van den Bergh H (1997) Photodynamic therapy for early squamous cell carcinomas of the esophagus, bronchi, and mouth with m-tetra(hydroxyphenyl) chlorin. *Arch Otolaryngol Head Neck Surg* **123**: 162–168

Star WM (1995) Diffusion theory of light transport. In *Optical-thermal Response of Laser-irradiated Tissue*, Welch AJ and van Gemert MJC (eds). Plenum: New York van Staveren HJ, Marijnissen JPA, Aalders MCG and Star WM (1995) Construction, quality control and calibration of spherical isotropic fibre-optic light diffusers. *Lasers Med Sci* **10**: 137–147

Wang Q, Altermatt HJ, Ris HB, Reynolds B, Stewart JCM, Bonnet R and Lim CK (1993) Determination of 5,10,15,20-tetra-(m-hydroxyphenyl)chlorin in tissues by high performance liquid chromatography. *Biomed Chromatogr* **7**: 155–157

Wilson BD, Mang TS, Stoll H, Jones C, Cooper M and Dougherty TJ (1992) Photodynamic therapy for the treatment of basal cell carcinoma. *Arch Dermatol* **128**: 1597–1601

© Cancer Research Campaign 1999
